# Comparative Developmental Neurotoxicity of Organophosphate Insecticides: Effects on Brain Development Are Separable from Systemic Toxicity

**DOI:** 10.1289/ehp.8828

**Published:** 2006-02-02

**Authors:** Theodore A. Slotkin, Edward D. Levin, Frederic J. Seidler

**Affiliations:** 1 Department of Pharmacology and Cancer Biology and; 2 Department of Psychiatry and Behavioral Sciences, Duke University Medical Center, Durham, North Carolina, USA

**Keywords:** acetylcholine, brain development, chlorpyrifos, diazinon, organophosphate insecticides, parathion

## Abstract

A comparative approach to the differences between systemic toxicity and developmental neurotoxicity of organophosphates is critical to determine the degree to which multiple mechanisms of toxicity carry across different members of this class of insecticides. We contrasted neuritic outgrowth and cholinergic synaptic development in neonatal rats given different organophosphates (chlorpyrifos, diazinon, parathion) at doses spanning the threshold for impaired growth and viability. Animals were treated daily on postnatal days 1–4 by subcutaneous injection so as to bypass differences in first-pass activation to the oxon or catabolism to inactive products. Evaluations occurred on day 5. Parathion (maximum tolerated dose, 0.1 mg/kg) was far more systemically toxic than was chlorpyrifos or diazinon (maximum tolerated dose, 1–5 mg/kg). Below the maximum tolerated dose, diazinon impaired neuritic outgrowth in the forebrain and brainstem, evidenced by a deficit in the ratio of membrane protein to total protein. Diazinon also decreased choline acetyltransferase activity, a cholinergic neuronal marker, whereas it did not affect hemicholinium-3 binding to the presynaptic choline transporter, an index of cholinergic neuronal activity. There was no m_2_-muscarinic acetylcholine receptor down-regulation, as would have occurred with chronic cholinergic hyper-stimulation. The same pattern was found previously for chlorpyrifos. In contrast, parathion did not elicit any of these changes at its maximum tolerated dose. These results indicate a complete dichotomy between the systemic toxicity of organophosphates and their propensity to elicit developmental neurotoxicity. For parathion, the threshold for lethality lies below that necessary for adverse effects on brain development, whereas the opposite is true for chlorpyrifos and diazinon.

Although some organophosphates are undergoing increasing scrutiny and restriction [U.S. Environmental Protection Agency (EPA) 2000, 2002] because of their propensity to elicit developmental neurotoxicity (Barone et al. 2000; Casida and Quistad 2004; Landrigan 2001; Rice and Barone 2000; Slotkin 2004), these compounds nevertheless still comprise 50% of all insecticide use worldwide, and exposure of the human population continues to be nearly ubiquitous (Casida and Quistad 2004). Originally, it was thought that the adverse effects on brain development reflected the same basic mechanism that underlies systemic toxicity, namely, cholinesterase inhibition and consequent cholinergic hyperstimulation (Mileson et al. 1998; Pope 1999). However, evidence accumulating over the past decade implicates a host of other mechanisms that depend instead upon the direct targeting of events specific to the developing brain (Barone et al. 2000; Pope 1999; Rice and Barone 2000; Slotkin 2004). Chlorpyrifos, the most-studied organo-phosphate, has been shown to disrupt the basic cellular machinery that controls the patterns of neural cell maturation and the formation and activity of synapses, exclusive of the effects on cholinesterase, which are mediated instead by its metabolite, chlorpyrifos oxon (Barone et al. 2000; Casida and Quistad 2004; Gupta 2004; Pope 1999; Qiao et al. 2002, 2003; Yanai et al. 2002). These mechanisms are likely to be shared by other organophosphates, but these have not been evaluated in detail (Abu-Qare and Abou-Donia 2001; Morale et al. 1998; Pope 1999; Qiao et al. 2001; Slotkin 1999, 2004; Whyatt et al. 2002).

A comparative approach to the differences between systemic toxicity and developmental neurotoxicity of organophosphates is critical to determine the degree to which multiple mechanisms of toxicity carry across different members of this class of insecticides. Although young animals are far more susceptible than adults to organophosphate-induced growth inhibition and lethality, there is a wide range over which disparate compounds elicit such effects. For example, parathion is far more systemically toxic to newborn rats than is chlorpyrifos, in part reflecting pharmacokinetic differences centering around the ontogeny of enzymes activating the parent compounds to the corresponding oxons, compared with the enzymes that break down the oxons to inactive metabolites (Atterberry et al. 1997; Padilla et al. 2000, 2004). The maximum tolerated doses of each agent correspond closely to the relative potencies toward cholinesterase inhibition and to the rate of recovery of cholinesterase activity, thus drawing a direct mechanistic connection of cholinergic hyperstimulation to overall systemic toxicity (Pope and Chakraborti 1992; Pope et al. 1991; Tang et al. 2003). In contrast, *in vitro* evaluations that bypass the pharmacokinetic differences suggest that chlorpyrifos is more potent toward inhibition of cell membrane function (Barber et al. 2001) and for eliciting cytotoxicity in immature neurons and glia (Monnet-Tschudi et al. 2000), despite the fact that parathion elicits greater cholinesterase inhibition (Zurich et al. 2000); indeed, physostigmine, a nonorganophosphate cholinesterase inhibitor, is far less effective in disrupting neural cell development *in vitro*, even at concentrations that completely block cholinesterase (Qiao et al. 2001; Zurich et al. 2000).

In the present study, we contrasted three organophosphates, chlorpyrifos, diazinon, and parathion, for their systemic toxicity compared with developmental neurotoxicity in the neonatal rat brain. We chose to administer each agent via subcutaneous injection in dimethyl sulfoxide (DMSO), a vehicle appropriate for water-insoluble agents and already known not to affect the corresponding measures of brain development (Qiao et al. 2001; Song et al. 1998; Whitney et al. 1995). The injection route also provides distinct advantages over oral gavage because it avoids the potential confounds of differential rates of gastrointestinal absorption between compounds or ages and first-pass effects on bioavailability. Parathion undergoes extremely high first-pass removal by the liver, reducing its oral bioavailability by more than 95% in the adult (Kramer and Ho 2002), effects that will therefore influence its relative toxicity at different developmental stages because of the rapid changes in the enzymes forming and destroying the oxon (Atterberry et al. 1997; Padilla et al. 2000, 2004). Furthermore, daily oral gavage and the associated repetitive stress are likely to exacerbate developmental toxicity and neurotoxicity (Colomina et al. 1995; Singer et al. 2002), including that associated with organophosphate administration (Shaikh et al. 2003); far less handling is required for a subcutaneous injection. Finally, the rat is an altricial species, so neurodevelopment in the immediate postnatal period corresponds to that in a second-to-early-third-trimester human fetus (Rodier 1988, 1995), in which exposure occurs via direct entry of the pesticides into the fetal circulation, rather than through oral, dermal, or inhalation routes.

For each agent, we evaluated doses spanning the threshold for the emergence of systemic toxicity as defined by growth impairment and decreased viability. These were then contrasted with four indices of neuronal development in the brainstem and forebrain that focus on two major classes of effects that have been characterized for chlorpyrifos: inhibition of neuritic outgrowth (Das and Barone 1999; Howard et al. 2005; Song et al. 1998) and the compromising of development of acetylcholine projections (Dam et al. 1999; Qiao et al. 2003; Richardson and Chambers 2005; Slotkin et al. 2001). First, we evaluated the ratio of membrane protein to total protein, which rises with the expansion of the cell membrane surface accompanying neuritic outgrowth (Qiao et al. 2003, 2004). Next, we evaluated the two biomarkers obligatory to the development of cholinergic neurons, activity of choline acetyltransferase (ChAT) and binding of hemicholinium-3 (HC3) to the cell membrane fraction, which assesses the expression of the high-affinity presynaptic choline transporter (Dam et al. 1999; Qiao et al. 2003, 2004; Richardson and Chambers 2005). ChAT, the enzyme that synthesizes acetylcholine, is a constitutive component of cholinergic nerve terminals and thus provides a measure of the development of cholinergic projections (Dam et al. 1999; Happe and Murrin 1992; Monnet-Tschudi et al. 2000; Qiao et al. 2003; Richardson and Chambers 2005; Slotkin et al. 2001). Unlike expression of ChAT, expression of the choline transporter is responsive to neuronal activity (Klemm and Kuhar 1979; Simon et al. 1976), so measurement of both parameters enables the distinction between effects on the development of innervation and those on synaptic activity. These markers have been used previously to characterize effects of chlorpyrifos on cholinergic systems in adult rats (Liu and Pope 1996, 1998) and to evaluate the immediate and delayed effects of postnatal chlorpyrifos exposure (Dam et al. 1999; Rhodes et al. 2004; Richardson and Chambers 2005; Slotkin et al. 2001). Finally, we also measured radioligand binding to the m_2_-muscarinic acetylcholine receptor (m_2_AChR), which is targeted by organophosphates in two distinct ways. First, the receptor typically undergoes down-regulation in the presence of excess acetylcholine, thus providing a time-integrated index of the degree of cholinergic hyperstimulation experienced by the developing brain after organophosphate exposure (Bushnell et al. 1993; Chakraborti et al. 1993; Ward and Mundy 1996). In addition, the oxons also bind directly to the m_2_AChR, affecting both its expression and its ability to elicit cellular signals (Howard and Pope 2002; Huff et al. 1994).

## Materials and Methods

### Animal treatments.

All experiments were carried out in accordance with the *Guide for the Care and Use of Laboratory Animals* (Institute of Laboratory Animal Resources 1996) as adopted and promulgated by the National Institutes of Health. Timed-pregnant Sprague-Dawley rats (Charles River, Raleigh, NC) were housed in breeding cages, with a 12-hr light/dark cycle and free access to food and water. On the day of birth, all pups were randomized and redistributed to the dams with a litter size of 9–10 to maintain a standard nutritional status; for treatment groups with high pup mortality rates (not used for neuro-chemical analyses), litter sizes were maintained in this range by combining groups of survivors. Chlorpyrifos, diazinon, and parathion (all from Chem Service, West Chester, PA) were dissolved in DMSO to provide consistent absorption (Whitney et al. 1995) and were injected subcutaneously in a volume of 1 mL/kg once daily on postnatal days (PND) 1–4; control animals received equivalent injections of the DMSO vehicle. For chlorpyrifos, we used daily doses of 1 mg/kg and 5 mg/kg, straddling the threshold for growth retardation and systemic toxicity (Campbell et al. 1997; Whitney et al. 1995). The lower dose produces neurotoxicity in developing rat brain with only 20% cholinesterase inhibition (Slotkin 1999, 2004; Song et al. 1997; Whitney et al. 1995), well below the 70% threshold necessary for symptoms of cholinergic hyperstimulation (Clegg and van Gemert 1999). This treatment thus resembles the nonsymptomatic exposures reported in pregnant women (De Peyster et al. 1993) and is within the range of expected fetal and childhood exposures after routine home application or in agricultural communities (Gurunathan et al. 1998; Ostrea et al. 2002). For diazinon and parathion, prior information on systemic toxicity using this vehicle and route was not available, so we evaluated a wider range of doses: 0.05–5 mg/kg for diazinon and 0.01–5 mg/kg for parathion. As shown in “Results,” just as for chlorpyrifos, the diazinon and parathion doses ranged from those with no discernible effect on growth or viability to those lying above the threshold for overt toxicity.

On PND5, one male and one female pup were selected from each of six litters in each treatment group and were used for neuro-chemical evaluations. Animals were decapitated, the cerebellum was removed, and the brainstem and forebrain were separated by a cut made rostral to the thalamus. Tissues were weighed, flash-frozen in liquid nitrogen, and maintained at −45°C until analysis.

### Assays.

Tissues were thawed in 79 volumes of ice-cold 10 mM sodium-potassium phosphate buffer (pH 7.4) and homogenized with a Polytron (Brinkmann Instruments, Westbury, NY). For ChAT activity (Lau et al. 1988), assays contained 60 mM sodium phosphate (pH 7.9), 200 mM NaCl, 20 mM choline chloride, 17 mM MgCl_2_, 1 mM EDTA, 0.2% Triton X-100, 0.12 mM physostigmine, 0.6 mg/mL bovine serum albumin, and 50 μM [^14^C]acetyl coenzyme A (specific activity, 60 mCi/mmol, diluted with unlabeled compound to 6.7 mCi/mmol; (PerkinElmer Life Sciences, Boston, MA). Samples were preincubated for 15 min on ice and transferred to a 37°C water bath for 30 min, and the reaction was terminated by placing the samples on ice. Labeled acetylcholine was then extracted and counted, and the activity was determined relative to total protein (Smith et al. 1985).

For measurements of [^3^H]HC3 binding (Vickroy et al. 1984), the cell membrane fraction was prepared by sedimenting an aliquot of the same tissue homogenate at 40,000 × *g* for 15 min. The membrane pellet was resuspended (Polytron) in the original volume of buffer and resedimented, and the resultant pellet was resuspended using a smooth glass homogenizer fitted with a Teflon pestle, in 10 mM sodium-potassium phosphate buffer (pH 7.4) and 150 mM NaCl. Radioligand binding was evaluated with 2 nM [^3^H]HC3 (specific activity, 125 Ci/mmol; PerkinElmer), with incubation for 20 min at room temperature, followed by rapid vacuum filtration onto Whatman GF/C filters (presoaked for 30 min with 0.1% polyethyleneimine in buffer). The nonspecific component was defined as radioligand binding in the presence of an excess concentration (10 μM) of unlabeled HC3 (Sigma Chemical Co., St. Louis, MO). Binding values were expressed relative to membrane protein. Similarly, for m_2_AChR binding, aliquots of the cell membrane fraction were incubated in 10 mM sodium-potassium phosphate buffer (pH 7.4) for 60 min at room temperature, using 1 nM [^3^H]AFDX384 (specific activity, 115 Ci/mmol; PerkinElmer) with or without 1 μM atropine (Sigma) to displace specific binding (Qiao et al. 2003).

The membrane protein:total protein ratio was evaluated from the measures of total tissue protein required for the ChAT assay and of membrane protein required for the ligand binding determinations.

### Data analysis.

Survival rates were compared with Fisher’s exact test using a one-tailed criterion because treatment with the organophosphates was expected to increase mortality. For parametric values, data were compiled as means and SEs. Because we evaluated multiple neurochemical variables that were all related to cholinergic synapses, the initial comparison was conducted by a global analysis of variance (ANOVA; two tailed) incorporating all the variables and measurements: treatment, sex, region (repeated measure within each animal), and effect (ChAT activity, HC3 binding, and m_2_AChR binding; repeated measure within each region). We identified significant interactions of treatment with sex and measure, and therefore data were subdivided for lower-order ANOVAs, followed by Fisher’s protected least significant difference test to evaluate individual treatments that differed from the corresponding control. Similarly, the membrane protein: total protein ratio was compared across treatments, regions, and sexes using multivariate ANOVA. In addition, dose–effect relationships were verified by multiple regression using the same three factors (dose, region, sex). Significance was assumed at *p* < 0.05 for all tests. For convenience, some data are presented as the percent change from control values, but statistical comparisons were conducted only on the original data. For reference, the corresponding control values are shown in Table 1.

## Results

**Figure 1:**
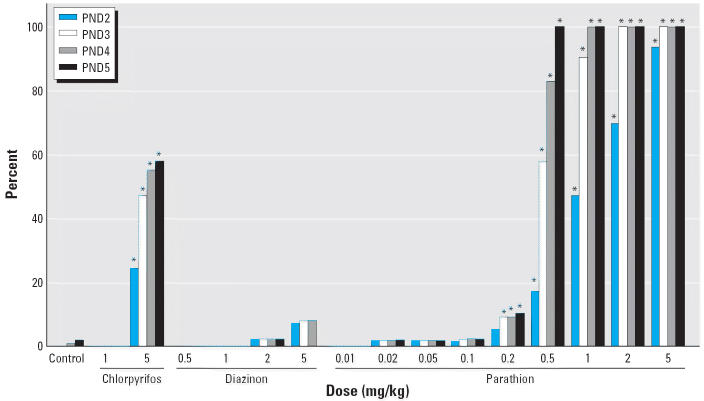
Effects of different organophosphates on mortality during daily treatment on PND1–4 and for up to 24 hr after the last dose. Data represent cumulative mortality obtained from a minimum of 60 animals in each treatment group at each age. Data for 5 mg/kg chlorpyrifos were compiled from previous results (Campbell et al. 1997; Whitney et al. 1995). *p < 0.05 compared with control, Fisher’s exact test.

In keeping with previous results (Campbell et al. 1997; Whitney et al. 1995), treatment with 1 mg/kg of chlorpyrifos on PND1–4 did not elicit any mortality, whereas raising the dose to 5 mg/kg produced a cumulative loss of more than half the animals by PND5 (Figure 1). For diazinon, doses of 0.5, 1, or 2 mg/kg had no effect on survival; raising the dose to 5 mg/kg resulted in the loss of < 10% of the neonates, an effect that did not achieve statistical significance but was obviously nearing the maximum tolerated dose. In contrast to chlorpyrifos or diazinon, parathion was much more lethal, causing significant mortality at doses > 0.1 mg/kg. At 0.2 mg/kg, the pattern for parathion resembled that of the highest dose of diazinon, with loss of a few animals at the initiation of treatment, without progressive increases in mortality after PND3. When the dose was raised to 0.5 mg/kg, however, all the animals given parathion died by PND5, and the same pattern was seen at 1, 2, and 5 mg/kg.

For neurochemical evaluations, we focused on treatments below the threshold for overt toxicity as defined by the mortality data: 1 mg/kg chlorpyrifos, 0.5–2 mg/kg diazinon, and 0.02–0.1 mg/kg parathion. At those doses, none of the treatments had a significant effect on body or brain region weights (data not shown). Nevertheless, there were significant effects on the ratio of membrane protein:total protein (*p* < 0.0001 for the main effect of treatment) and for the three measures related to cholinergic synaptic function (*p* < 0.05 for treatment × sex; *p* < 0.03 for treatment × measure). Because of the significant interactions with sex and measure, results were separated for the different measures, and treatment and sex effects were evaluated across the two brain regions. Results for chlorpyrifos have been published previously (Dam et al. 1999; Song et al. 1997), so here we focus on diazinon and parathion.

**Figure 2:**
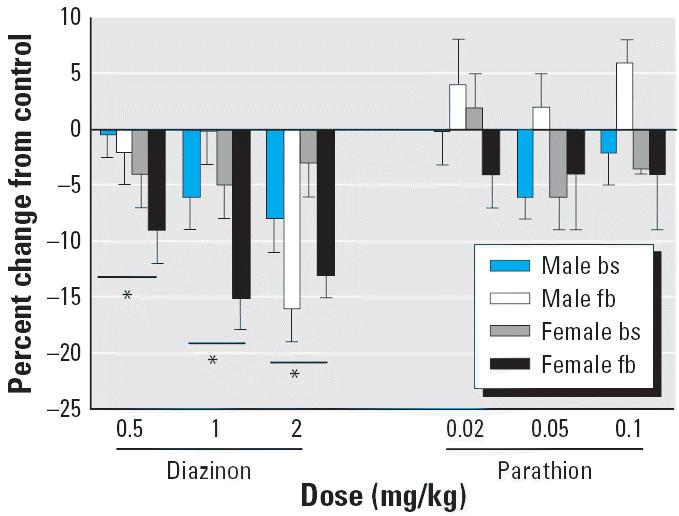
Effects of different doses of diazinon and parathion on the membrane protein:total protein ratio in brainstem (bs) and forebrain (fb), assessed on PND5 and presented as the percent change from the corresponding control values (Table 1). ANOVA across all treatments, both regions and both sexes: main treatment effect, *p* < 0.0001. *Significantly different (*p* < 0.05) from corresponding control values; statistical significance for individual regions or sexes was not determined because of the absence of treatment × region and treatment × sex interactions.

Diazinon treatment produced a dose-dependent decrease in the membrane protein:total protein ratio that was statistically significant even at 0.5 mg/kg (Figure 2); the dose–effect relationship was confirmed by multiple regression incorporating the factors of dose, region, and sex, demonstrating a significant correlation with dose (*p* < 0.0001). There were no significant distinctions between males and females or between the brainstem and forebrain (no treatment × sex or treatment × region interaction). In contrast, parathion treatment up to the maximum tolerated dose of 0.1 mg/kg had no discernible effect on this index.

**Figure 3:**
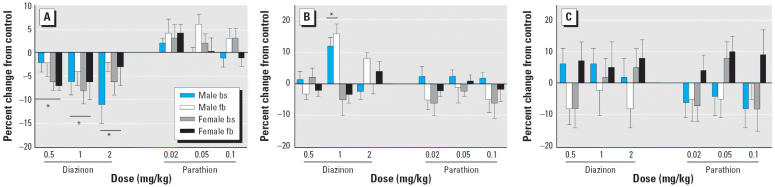
Effects of different doses of diazinon and parathion on markers of cholinergic synaptic development in brainstem (bs) and forebrain (fb), assessed on PND5. (*A*) ChAT activity (ANOVA: treatment, *p* < 0.0001). (*B*) m_2_AChR receptor binding (ANOVA: treatment, *p* < 0.03; treatment × sex, *p* < 0.0002; male, *p* < 0.0001. (*C*) HC3 binding to the high-affinity presynaptic choline transporter (ANOVA: treatment × sex × region, *p* < 0.05). Data are presented as the percent change from the corresponding control values (Table 1). *Significantly different (*p* < 0.05) from corresponding control values; statistical significance for individual regions or sexes was determined only where there were corresponding treatment × region or treatment × sex interactions.

Among the three cholinergic synaptic markers, the most consistent effect was on ChAT activity (Figure 3A). As was seen for the membrane protein:total protein ratio, diazinon elicited a dose-dependent deficit in ChAT (*p* < 0.003 for the correlation of ChAT with dose in multiple regression), whereas parathion was ineffective up to its maximum tolerated dose. We did not observe any significant down-regulation of m_2_AChRs with either diazinon or parathion, and in fact, the intermediate dose of diazinon (1 mg/kg) elicited a significant increase in males that was no longer evident when the dose was raised to 2 mg/kg, still below the threshold for significant mortality (Figure 3B). There were no discernible effects on HC3 binding with any of the treatments (Figure 3C). The inherently higher variability of HC3 binding decreases the likelihood of detecting significant differences of the magnitude of those found for the other cholinergic markers; nevertheless, the lack of significance for the HC3 marker was statistically distinguishable from the decrement in ChAT (*p* < 0.05 for the treatment × measure interaction).

## Discussion

Chlorpyrifos exposure during the perinatal period is known to evoke deficits in neuritic outgrowth, specifically including the targeting of cholinergic projections (Dam et al. 1999; Das and Barone 1999; Howard et al. 2005; Qiao et al. 2002, 2003; Slotkin et al. 2001; Song et al. 1998). Indeed, administration of 1 mg/kg on PND1–4, a regimen below the threshold for impairment of growth or viability, elicits only 20% inhibition of cholinesterase (Song et al. 1997), well below the 70% threshold for symptoms of cholinergic hyper-stimulation (Clegg and van Gemert 1999). Nevertheless, as shown previously (Dam et al. 1999), as early as 1 day after neonatal chlorpyrifos exposure (PND5), there is a shortfall in ChAT, the constitutive marker of cholinergic projections, without affecting HC3 binding, the index of synaptic activity. At this dose, down-regulation of m_2_AChRs does not occur, and m_1_AChRs decrease by only 10% (Song et al. 1997), consistent with only a small degree of cholinesterase inhibition. The initial deficits in the development of cholinergic projections lead to the subsequent emergence of abnormalities of cholinergic innervation, substantial deficits in cholinergic synaptic activity, and related behavioral anomalies in adolescence and adulthood (Dam et al. 2000; Levin et al. 2001; Slotkin 1999, 2004; Slotkin et al. 2001). The effects of chlorpyrifos at its maximum tolerated dose of 1 mg/kg can thus serve as a benchmark for parallel comparisons of the effects of diazinon and parathion as evaluated in the present study.

With *in vitro* models or lower organisms, diazinon, like chlorpyrifos, has been shown to interfere with neural cell replication and differentiation (Axelrad et al. 2003; Morale et al. 1998; Qiao et al. 2001; Shin et al. 2001). Here, in neonatal rats, diazinon exhibited less systemic toxicity than chlorpyrifos, with no growth impairment or significant loss of viability up to a dose of 5 mg/kg. Nevertheless, at exposures well below the maximum tolerated dose, diazinon reduced the membrane protein:total protein ratio, a result in keeping with restriction of neuritic outgrowth. Also like chlorpyrifos, diazinon produced a deficit in ChAT, consistent with targeting of the development of cholinergic projections, without discernible effect on HC3 binding, the index of impulse activity. However, it should be noted that the greater variability of HC3 binding renders it problematic to detect small changes, so an effect on cholinergic synaptic activity cannot be ruled out. Nevertheless, it is notable that the same pattern, decreased ChAT without a change in HC3 binding, is seen at the same early stage after neonatal chlorpyrifos treatment (Dam et al. 1999), and deficits in HC3 binding do not emerge until much later in development (Slotkin et al. 2001). Accordingly, it would be valuable to carry out longitudinal studies of cholinergic synaptic function and related behavioral anomalies after neonatal diazinon exposure, parallel to those already completed for chlorpyrifos (Levin et al. 2001; Slotkin 2004; Slotkin et al. 2001). As was also found with chlorpyrifos (Song et al. 1997), diazinon treatment affected neuritic outgrowth and ChAT without down-regulating m_2_AChR binding, in keeping with the absence of signs of cholinergic hyperstimulation and consistent with mechanisms unrelated to cholinesterase inhibition. Indeed, the only change was a significant increase at 1 mg/kg that was lost when the dose was raised to 2 mg/kg. The biphasic pattern has also been noted previously with chlorpyrifos (Levin et al. 2002; Qiao et al. 2002), and there are two distinct possibilities for this hormetic response. First, a small degree of cholinergic stimulation can be promotional for neural cell differentiation because of the neurotrophic role of acetylcholine (Lauder and Schambra 1999), whereas that effect would likely be offset when the dose is raised closer to the threshold for systemic toxicity (Qiao et al. 2002). Alternatively, the ability of the organophosphates and their oxons to bind to the m_2_AChR and interfere with its function (Howard and Pope 2002; Huff et al. 1994) would be likely to elicit compensatory up-regulation of receptor expression, which would then be offset by down-regulation consequent to cholinesterase inhibition as the dose is raised.

The effects of parathion stand in stark contrast to those of chlorpyrifos and diazinon. As found in previous work (Atterberry et al. 1997; Liu et al. 1999; Padilla et al. 2004; Pope and Chakraborti 1992; Pope et al. 1991; Tang et al. 2003), parathion was far more potent in eliciting systemic toxicity, with a threshold for lethality at 0.2 mg/kg, fully an order of magnitude below those for the other two organo-phosphates. We administered each agent by subcutaneous injection, so first-pass differences in hepatic activation to the corresponding oxon or catabolism to inactive products clearly cannot account for these differences. Because the maximum tolerated dose is directly related to the degree of cholinesterase inhibition (Pope and Chakraborti 1992; Pope et al. 1991; Tang et al. 2003), our results provide a framework for evaluating the relative contributions of cholinesterase inhibition versus other mechanisms in the developmental neurotoxicity of organophosphates. If the effects of parathion at its maximum tolerated dose parallel those of chlorpyrifos and diazinon at their maximum tolerated doses, which are much higher, then cholinesterase inhibition is likely to be the most important factor; on the other hand, if these effects are unrelated to cholinesterase inhibition and resultant systemic toxicity, then the lower dose of parathion should be relatively ineffective in producing developmental neurotoxicity. Our results clearly point to the latter outcome: Parathion administration up to the maximum tolerated dose of 0.1 mg/kg had no discernible effect on the membrane protein:total protein ratio or on ChAT activity. Our results do not mean that parathion is incapable of eliciting developmental neurotoxicity, but rather that the dose required for effects on brain development exceeds the threshold for overt systemic toxicity, a situation opposite that for chlorpyrifos or diazinon. In support of this interpretation, higher doses of parathion administered to pregnant rats throughout gestation do affect ChAT but only when the dose is sufficiently high to elicit clear signs of maternal toxicity and down-regulation of mAChR binding (Gupta et al. 1985); similarly, paraoxon administration over a prolonged postnatal period, at doses that decrease weight gain and viability, impairs the development of neuritic projections (Santos et al. 2004), precisely the effects seen for chlorpyrifos and diazinon at exposures below the maximum tolerated dose. *In vitro* test systems also suggest that chlorpyrifos is inherently more toxic to the developing brain than is parathion (Barber et al. 2001; Monnet-Tschudi et al. 2000), the opposite of their relationship for cholinesterase inhibition and systemic toxicity. Obviously, future work needs to address the specific mechanisms that determine the separable effects of the different organophosphates on neurodevelopment. It is unlikely that these reside in simple physico-chemical characteristics such as lipid solubility, neither for systemic toxicity nor for developmental neurotoxicity; the latter is not surprising, considering that the blood–brain barrier is incomplete in the neonate and in any case is not an issue for penetration of highly lipophilic compounds such as the organophosphates (Saunders and Møllgard 1984). Chlorpyrifos, diazinon, and parathion are all highly lipid soluble (partition coefficients in the thousands), with a rank order of chlorpyrifos >> parathion ≈ diazinon (Bowman and Sans 1979; Davies et al. 1975; Sartorelli et al. 1998; Sunshine 1969), yet the developmental effects of chlorpyrifos and diazinon were similar, whereas those for parathion were different.

In conclusion, different organophosphates share the ability to elicit developmental neurotoxicity converging on a common set of events, including impaired neuritic outgrowth and impaired development of characteristics that are critical to the phenotypic differentiation and function of cholinergic neurons. However, these effects are entirely disjunct from systemic toxicity, which instead largely reflects cholinesterase inhibition. In fact, the developmental neurotoxicity of parathion emerges only at doses exceeding the threshold for overt toxicity, whereas the corresponding effects of chlorpyrifos and diazinon are apparent at exposures below the maximum tolerated dose. Our findings thus emphasize the need to examine fetal and neonatal neurotoxicity of multiple organophosphates in a fashion similar to that already conducted for chlorpyrifos, as well as reinforcing the need for replacement of the “gold standard,” cholinesterase inhibition, with biomarkers of neural development, the true end points for the developmental neurotoxicity of organophosphates.

## Figures and Tables

**Table 1:** Neurochemical parameters in brain regions of controls.

	Brainstem		Forebrain	
Measure	Male	Female	Male	Female
Membrane protein:total protein (%)	28.2 ± 0.7	27.1 ± 0.5	25.0 ± 0.8	27.0 ± 0.8
ChAT (pmol/min/mg protein)	188 ± 4	191 ± 2	58 ± 1	61 ± 1
m_2_AChR binding (fmol/mg protein)	167 ± 4	174 ± 4	263 ± 6	258 ± 7
HC3 binding (fmol/mg protein)	28 ± 1	29 ± 1	15.9 ± 0.9	13.8 ± 0.7
